# Comparison of risk allele frequencies of single nucleotide polymorphisms associated with age-related macular degeneration in different ethnic groups

**DOI:** 10.1186/s12886-021-01830-9

**Published:** 2021-02-22

**Authors:** Hyun-Tae Shin, Byung Woo Yoon, Je Hyun Seo

**Affiliations:** 1 Veterans Medical Research Institute, Veterans Health Service Medical Center, Jinhwangdo-ro 61-gil 53, Gangdong-gu, Seoul, 05368 South Korea; 2 Department of Dermatology, Inha University School of Medicine, Inha-ro 100, Michuhol-gu, Incheon, 22212 South Korea; 3 Division of Oncology, Department of Internal Medicine, Inje University Seoul Paik Hospital, Mareunnae-ro 9, Jung-gu, Seoul, 04551 South Korea

**Keywords:** Age-related macular degeneration, Allele frequency, Single nucleotide polymorphism, Genetic risk scores, Prevalence

## Abstract

**Background:**

The prevalence of age-related macular degeneration (AMD) varies from 6.8 to 18.3% for all forms of AMD and from 0.6 to 2.6% for late AMD according to race, suggesting the existence of genetic differences among races. The purpose of this study was to determine the genetic causes of differences in the prevalence of AMD among individuals of different races.

**Methods:**

We collected 138 AMD-associated single nucleotide polymorphisms (SNPs) from a genome-wide association studies catalog. Their population-level allele frequencies were derived based on the 1000 Genomes Project and Korean Reference Genome Database. We used Fisher’s exact tests to assess whether the effect allele at a given SNP was significantly enriched or depleted in the database.

**Results:**

European, American, and South Asian populations showed similar heatmap patterns, whereas East Asian, and Korean populations had distinct patterns. Korean populations exhibited patterns that were different from those of the other groups; rs5754227 (*SYN3*), rs1626340 (*TGFBR1/COL15A1*), rs3750846(*ARMS2/HTRA1*), and rs9564692 (*B3GALTL*) were enriched, whereas rs2230199 (*C3*) and rs73036519 (*EXOC3L2/MARK4*) were depleted in Koreans; these SNPs are associated with late AMD. The genetic risk score calculated from allele frequencies was not less in East Asians than in Europeans.

**Conclusion:**

The prevalence of AMD is lower in Asians than in Europeans. However, our study showed that genetic risk scores in East Asians were similar to those in Europeans, which may explain why the global projected number of people with AMD by 2040 is in largest for East Asians, including Koreans.

**Supplementary Information:**

The online version contains supplementary material available at 10.1186/s12886-021-01830-9.

## Background

Age-related macular degeneration (AMD) is the leading cause of visual impairment in developed countries [1, 2], particularly in people older than 60 years. AMD includes early AMD and late AMD; early AMD is characterized by drusen and/or pigmentary changes [3, 4], whereas late AMD is can show two subtypes, i.e., geographic atrophy (GA) and choroidal neovascularization (CNV). According to a study by Wong et al., the global prevalence of all types of AMD is approximately 8.7% worldwide [5]. In Europe, the prevalence of early AMD increases from 3.5% in those aged 55–59 years to 17.9% in those aged 85 years; for late AMD, the prevalence increases from 0.1 to 9.8% [6]. For Asian AMD, the prevalence rates of early and late AMD were found to range from 1.4 to 17.3% and 0.1 to 7.3%, respectively, with a higher prevalence in elderly groups [7, 8]. Importantly, the number of people affected is expected to increase dramatically during the coming decades as Asian populations age, and it is estimated that there will be more Asians with AMD than in the rest of the world combined by 2040 [2].

AMD (both early and late) is common among non-Hispanic white and Hispanic populations, but is less common in non-Hispanic black populations; in contrast, late AMD is less common in Hispanics than in non-Hispanic whites [9–12]. These differences suggest that genetic factors could be important determinants of AMD. In recent years, large consortiums using genome-wide association studies (GWASs) have revealed 52 independently associated variants spanning 34 loci, including the complement factor H (*CFH*), complement component 3 (C3), *ARMS2/HTRA1*, and complement factor I (*CFI*) genes [13]. Additional meta-analyses have shown that several single nucleotide polymorphisms (SNPs) and variants located in extracellular matrix-related genes, such as *COL15A1*, *COL8A1*, *MMP19*, and *CTRB2/CTRB1*, were significantly associated with late AMD [13–15]. Thus, by combining these results and data from the GWAS catalog of the National Human Genome Research Institute-European Bioinformatics Institute (NHGRI-EBI), it may be possible to identify SNPs related to any AMD (i.e., both early and late) and to late AMD specifically.

Accordingly, in this study, we compiled a comprehensive set of SNPs associated AMD and assessed differences in allele frequencies in various populations by utilizing phase 3 data from the 1000 Genome Project [16], which identifies genetic variants among 26 worldwide populations. Because this database does not include Korean genome data, it was possible to assess allele frequency pattern-related AMD in Koreans using the Korean Reference Genome Database (KRGDB) [17], which consists of 1722 whole-genome sequencing data from healthy Koreans produced by the Korea National Institute of Health in 2016. The rationale for conducting this study was to apply whole-genome sequencing data of healthy subjects to aging-related disease, which suggested by previous researchers [18]. Hence, we aimed to gain insights into genetic causes of allele frequencies differences among races for SNPs related to AMD and to compare composite genetic risk scores and polygenetic risk scores using SNPs related to AMD and AMD prevalence for different ethnic groups, including Koreans.

## Methods

### Ethical considerations

This study was approved and monitored by the Institutional Review Board (IRB) of the Veterans Health Service Medical Center, Korea (IRB no. 2019–07-008).

### Comparison of AMD-related SNPs in the global population and east Asians

AMD can be classified as early or late AMD. Early AMD is characterized by yellow subretinal deposits or retinal pigment epithelial irregularities, whereas late AMD is characterized by leakage of the choroidal neovascular membrane fluid or blood into the subretinal space or GA. “Any AMD” included both types of AMD. We searched the NHGRI-EBI GWAS catalog (https://www.ebi.ac.uk/gwas/home, December 2019) for SNPs that were associated with AMD traits (EFO_0001365). The catalog included 28 studies, which revealed 232 associations. After eliminating repetitive SNPs and removal of information not found in the 1000 Genome Projects database, 138 AMD-associated SNPs from the GWAS catalog were used for analysis of allele frequencies associated with any AMD (**Supplementary Tables** **1****and** **2**).

Among SNPs associated with AMD-related traits, we determined AMD risk by examining beta-coefficients and odds ratios for the effect allele. We also read the text descriptions in the primary GWAS reports. The details and advantages of this method have been described elsewhere [19]. Briefly, the population-level allele frequencies of SNPs were derived based on the 1000 Genomes Project phase 3 (*n* = 2504) and KRGDB (*n* = 1722). The 1000 Genomes Project surveyed genetic variations among 2504 individuals from 26 worldwide populations, grouped into African, East Asian, European, South Asian, and American populations based on their geographical locations and ancestries [16]; the data were downloaded from ftp://ftp.1000genomes.ebi.ac.uk/vol1/ftp/release/20130502/ (last accessed: January 15, 2020). The variant coordinates were based on the human genome assembly GRCh37. Because the East Asian data in the 1000 Genomes Project did not include data from Korean populations, we compared the data from five continents and East Asian countries in the 1000 Genome Projects with data extracted from KRGDB, which included whole-genome sequencing data for 1722 Korean individuals [17]. Data on the population frequencies of the SNPs were downloaded from the web-based database (http://152.99.75.168:9090/KRGDB/menuPages/download.jsp/, last accessed: January 15, 2020). For comparison of the distributions of individual risk alleles of the Korean population, individual genotyping results from the second phase of KRGDB (*n* = 1099) were obtained from the National Human Resource Bank of Korea.

### Comparison of SNPs related to late AMD in the global population and east Asians

Late AMD in this study included CNV and GA. Although a previous study revealed the SNPs related to each type of late AMD, it is difficult to select only one phenotype because the SNPs are often related to both CNV and GA [13]. SNPs related to late AMD are shown in Table 1, and these data were obtained from overlapping SNPs from GWAS catalog data and a study by the International AMD Genomics Consortium [13, 14]. The population-level allele frequencies of these SNPs were derived as described above.

**Table 1:** Effect allele frequencies (EAFs) of 31 late age-related macular degeneration related single nucleotide polymorphisms in continental groups, including Koreans

SNP ID	Chr	Position	Function	Ref allele	Alt allele	Nearby/containing Gene	Global EAF	AMR EAF	AMR log_**10**_ ***P***	AFR EAF	AFR log_**10**_ ***P***	EAS EAF	EAS log_**10**_ ***P***	SAS EAF	SAS log_**10**_ ***P***	EUR EAF	EUR log_**10**_ ***P***	KOR EAF	KOR log_**10**_ ***P***
rs10033900	chr4	110,659,067	intergenic	T	C	CFI	0.49	0.53	1.06	0.66	27.44	0.39	−8.01	0.31	−23.92	0.54	2.30	0.34	−43.57
rs10781182	chr9	76,617,720	intergenic	T	G	MIR6130/RORB	0.46	0.54	3.26	0.15	−101.56	0.49	1.04	0.53	3.66	0.70	42.77	0.41	−4.43
rs10922109	chr1	196,704,632	intronic	C	A	CFH	0.51	0.55	1.06	0.56	2.79	0.44	−4.05	0.56	2.15	0.43	−5.16	0.47	−3.33
rs11080055	chr17	26,649,724	intronic	A	C	TMEM97/VTN	0.58	0.58	0.00	0.61	1.23	0.72	16.01	0.48	−7.41	0.49	−6.53	0.76	64.55
rs1142	chr7	104,756,326	downstream	C	T	KMT2E/SRPK2	0.31	0.33	0.31	0.27	−2.23	0.37	3.55	0.23	−5.81	0.36	2.56	0.34	1.89
rs11884770	chr2	228,086,920	ncRNA_intronic	T	C	COL4A3	0.67	0.72	1.62	0.49	−31.04	0.78	11.41	0.73	3.30	0.71	1.76	0.76	20.03
rs12019136	chr19	5,835,677	intronic	G	A	C3	0.15	0.05	−14.85	0.32	39.45	0.14	−0.34	0.14	−0.31	0.04	−24.78	0.08	−23.98
rs12357257	chr10	24,999,593	intronic	G	A	ARHGAP21	0.14	0.17	1.07	0.11	−2.37	0.05	−15.48	0.16	0.79	0.25	15.64	0.03	−65.27
rs13081855	chr3	99,481,539	ncRNA_intronic	G	T	COL8A1/FILIP1L	0.07	0.06	−0.31	0.03	−4.92	0.05	−1.23	0.10	3.36	0.10	3.01	0.03	−15.11
rs1626340	chr9	101,923,372	intergenic	G	A	TGFBR1/COL15A1	0.29	0.25	−1.07	0.29	0.00	0.46	23.83	0.24	−2.47	0.21	−6.58	0.51	95.30
rs181705462	chr6	31,947,027	intronic	G	T	C2/CFB/SKIV2L	0.01	0.01	0.00	0.01	0.18	0.00	−1.52	0.02	0.66	0.01	0.38	0.00	−4.86
rs2043085	chr15	58,680,954	intergenic	T	C	AQP9;LIPC	0.49	0.62	8.95	0.36	−16.28	0.50	0.23	0.44	−2.15	0.63	15.08	0.48	−0.30
rs2230199	chr19	6,718,387	exonic	G	C	C3	0.09	0.10	0.31	0.03	−11.70	0.00	−30.53	0.10	0.66	0.22	28.35	0.00	−97.67
rs2740488	chr9	107,661,742	intronic	A	C	ABCA1	0.33	0.28	−1.62	0.43	10.40	0.24	−7.72	0.38	2.29	0.25	−6.05	0.26	−12.12
rs3138141	chr12	56,115,778	ncRNA_intronic	C	A	RDH5/CD63/MMP19	0.15	0.11	−1.86	0.01	−61.21	0.12	−1.76	0.38	53.57	0.19	2.57	0.07	−32.36
rs3750846	chr10	124,215,565	intronic	T	C	ARMS2/HTRA1	0.29	0.25	−1.07	0.26	−1.44	0.41	12.35	0.34	2.47	0.19	−10.25	0.42	32.52
rs429358	chr19	45,411,941	exonic	T	C	APOE	0.15	0.10	−2.81	0.27	21.51	0.09	−7.38	0.09	−6.61	0.16	0.37	0.09	−14.75
rs55975637	chr3	99,419,853	ncRNA_intronic	G	A	COL8A1	0.08	0.08	−0.04	0.05	−2.62	0.06	−1.52	0.09	0.40	0.12	4.31	0.03	−20.71
rs570618	chr1	196,657,064	intronic	T	G	CFH	0.78	0.78	0.00	0.81	1.71	0.95	43.09	0.71	−4.95	0.64	−18.54	0.92	72.90
rs5754227	chr22	33,105,817	intronic	T	C	SYN3/TIMP3	0.37	0.39	0.31	0.56	33.60	0.61	42.65	0.12	−57.77	0.13	−53.79	0.67	167.56
rs61818925	chr1	196,815,450	intergenic	T	G	CFHR1	0.59	0.52	−2.60	0.72	17.39	0.41	−24.37	0.69	7.77	0.58	−0.26	0.50	−15.15
rs61985136	chr14	68,769,199	intronic	C	T	RAD51B	0.45	0.60	11.79	0.13	−110.82	0.45	0.00	0.63	23.38	0.58	12.87	0.42	−2.45
rs62247658	chr3	64,715,155	ncRNA_intronic	C	T	ADAMTS9-AS2	0.28	0.29	0.15	0.13	−30.69	0.24	−1.92	0.22	−3.58	0.56	60.51	0.20	−15.79
rs6565597	chr17	79,526,821	intronic	C	T	NPLOC4/TSPAN10	0.24	0.23	−0.15	0.16	−9.47	0.21	−1.28	0.26	0.66	0.36	13.46	0.21	−2.48
rs67538026	chr19	1,031,438	intronic	C	T	CNN2	0.38	0.33	−1.54	0.15	−60.48	0.52	15.07	0.49	8.86	0.45	4.28	0.50	24.75
rs72802342	chr16	75,234,872	intergenic	C	A	CTRB2/CTRB1	0.06	0.06	0.00	0.01	−21.05	0.13	11.80	0.04	−1.35	0.08	1.56	0.11	15.95
rs73036519	chr19	45,748,362	intergenic	G	C	EXOC3L2/MARK4	0.23	0.17	−2.80	0.36	19.67	0.00	−98.90	0.29	3.66	0.29	4.05	0.00	− 284.04
rs7803454	chr7	99,991,548	intronic	C	T	PILRB/PILRA	0.09	0.11	1.00	0.03	−16.28	0.03	−11.57	0.11	1.42	0.19	18.37	0.02	−37.14
rs8135665	chr22	38,476,276	intronic	C	T	SLC16A8	0.25	0.31	2.43	0.37	16.33	0.14	−14.06	0.20	−2.76	0.22	−1.32	0.10	−65.11
rs943080	chr6	43,826,627	intergenic	C	T	VEGFA	0.67	0.52	−12.42	0.83	30.61	0.73	3.60	0.67	0.00	0.51	−20.22	0.67	0.14
rs9564692	chr13	31,821,240	intronic	C	T	B3GALTL	0.50	0.51	0.15	0.45	−2.79	0.71	33.55	0.54	1.42	0.33	−22.06	0.73	101.51

*Chr* chromosome, *EAF* effect allele frequency, *ref. allele* reference allele, *alt allele* alterative allele, *AMR* Americans, *AFR* Africans, *EAS* East Asians, *SAS* South Asians, *EUR* Europeans, *KOR* Koreans *P*-value: adjusted Fischer’s test assuming 138 hypotheses, statistical significance was set at *P <* 0.05

### Calculation of genetic risk scores using SNPs related to any AMD and late AMD

To compare the composite genetic risk of AMD, we adopted the following equation described by Mao et al [19]:

$$ Genetic\ risk\ score=\frac{\sum_{i=1}^I\  Xi}{2I} $$

where “I” refers to the number of AMD-related SNPs, and “Xi” refers to copies of risk alleles (Xi ∈ {0,1,2}) at the i^th^ SNP. In one extreme case, if a person has two copies of the risk allele at each AMD-related SNP, then the person’s risk score was set as 1. In contrast, if a person has no copies of risk alleles at any AMD-related SNP, then the person’s risk score was set as 0. A person with a composite score of 1 had the highest possible genetic risk for AMD, whereas a person with a score of 0 had the lowest possible genetic risk. If copies of effect alleles (0/1/2) were randomly assigned to each SNP, the expected value of the risk score was set at 0.5. In addition, polygenic risk score (PRS) examine the cumulative effect of genetic variants on a disease or trait by aggregating the individual genetic effects into a single measurement [20]. To compare the PRS, we adopted the following equation:

$$ Polygenic\ risk\ score=\frac{\sum_{i=1}^I\ \beta iXi}{2I} $$

Where "*βi* ” refers to an average odd ratio of the i^th^ SNP reported in the GWAS studies.

SNPs with frequency differences of more than 10% between the total (*n* = 1722) and second phase (*n* = 1099) data of KRGDB were excluded from the genetic score calculation. We used the average composite genetic risk scores for populations to determine correlations with country-wise AMD prevalence data. For subgroup analysis of late AMD, the relationships between the composite genetic risk score using late AMD-related SNPs and continental-wise late AMD prevalence data were analyzed. Because the prevalence of AMD is affected by age, the prevalence was analyzed for individuals ages greater than or equal to 40 years old or greater than or equal to 65 years old. The prevalence data for any AMD and late AMD were evaluated based in the results of studies on Europeans [2, 6, 10, 21], East Asians [2, 7, 21, 22], Americans [2, 10] and Koreans [8, 23, 24]. Studies on prevalence among Japanese people were sufficient; thus, the prevalence data for Japanese populations were used to represent East Asians. Data on the prevalence of AMD in Africans and South Asians were limited; therefore, these populations were not included in this analysis.

### Data analyses

We used Fisher’s exact test to assess whether the effect allele at a given SNP was significantly enriched or depleted compared with the global population frequency in the 1000 Genomes Project database. Since we compared 138 SNPs, we calculated adjusted *P* values assuming 138 hypotheses and the *P* values were first log_10_-transformed. In the heatmap generated to visualize allele enrichment or depletion patterns in different populations, red and purple colors indicated higher and lower frequencies than the global average, respectively. If the effect allele of an SNP was enriched in a population, then the negative of log_10_ of the enrichment *P* value (a positive number) was used to represent the SNP associated with that population in the heatmap. In contrast, if the allele of an SNP was depleted in a population, the value of log_10_ of the depletion *P* value (a negative number) was used to represent the SNP for that population in the heatmap. Statistical analyses were performed using R software version 3.6.0 (R Foundation, Vienna, Austria). Statistical significance was set at *P <* 0.05.

## Results

### Patterns of any AMD-related SNPs in the global population and in east Asians

**Fig. 1:**
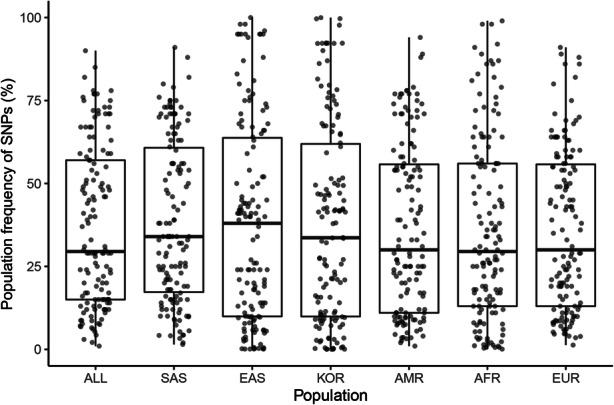
Comparison of the frequency of age-related macular degeneration (AMD)-related single nucleotide polymorphisms (SNPs) according to super population. AMR: American, EUR: European, SAS: South Asian, AFR: African, EAS: East Asian, KOR: Korean

We collected 232 AMD-associated SNPs from 24 GWASs from the NHGRI-EBI GWAS catalog. The SNPs were identified in 17 European populations, four East Asian populations, and three South Asian populations; three studies were performed in mixed ethnic populations. Clearly, populations other than Europeans were understudied, particularly African and American populations. However, there were no significant differences in the SNP frequencies among these populations (Fig. 1). This result suggested that many SNPs found in Europeans were also applicable to other populations. After collecting 138 AMD-associated SNPs, we determined the effect allele frequencies (EAFs) for each of the continental groups and for Koreans based on genotype information from the 1000 Genomes Project and KRGDB (**Supplementary Table S****1**). Heatmap analysis showed how significantly the effect allele was enriched or depleted across the Korean and continental groups (**Supplementary Fig.** **1**). For Koreans, 55 AMD-related SNPs were significantly enriched, 74 SNPs were depleted, and nine SNPs were similar to the global EAFs. Additionally, heatmap analysis enabled visualization of the proportion of effect alleles in each continental group compared with the global average. For example, rs5754227 (*SYN3*), rs1626340 (*TGFBR1/COL15A1*), rs3750846 (*ARMS2/HTRA1*), and rs9564692 (*B3GALTL*) were enriched, whereas rs2230199 (*C3*) and rs73036519 (*EXOC3L2/MARK4*) were depleted in Koreans. The hierarchical clustering tree showed differences among the populations; Europeans, Americans, and South Asians were in one cluster, whereas Africans, East Asians, and Koreans were in another cluster.

**Fig. 2:**
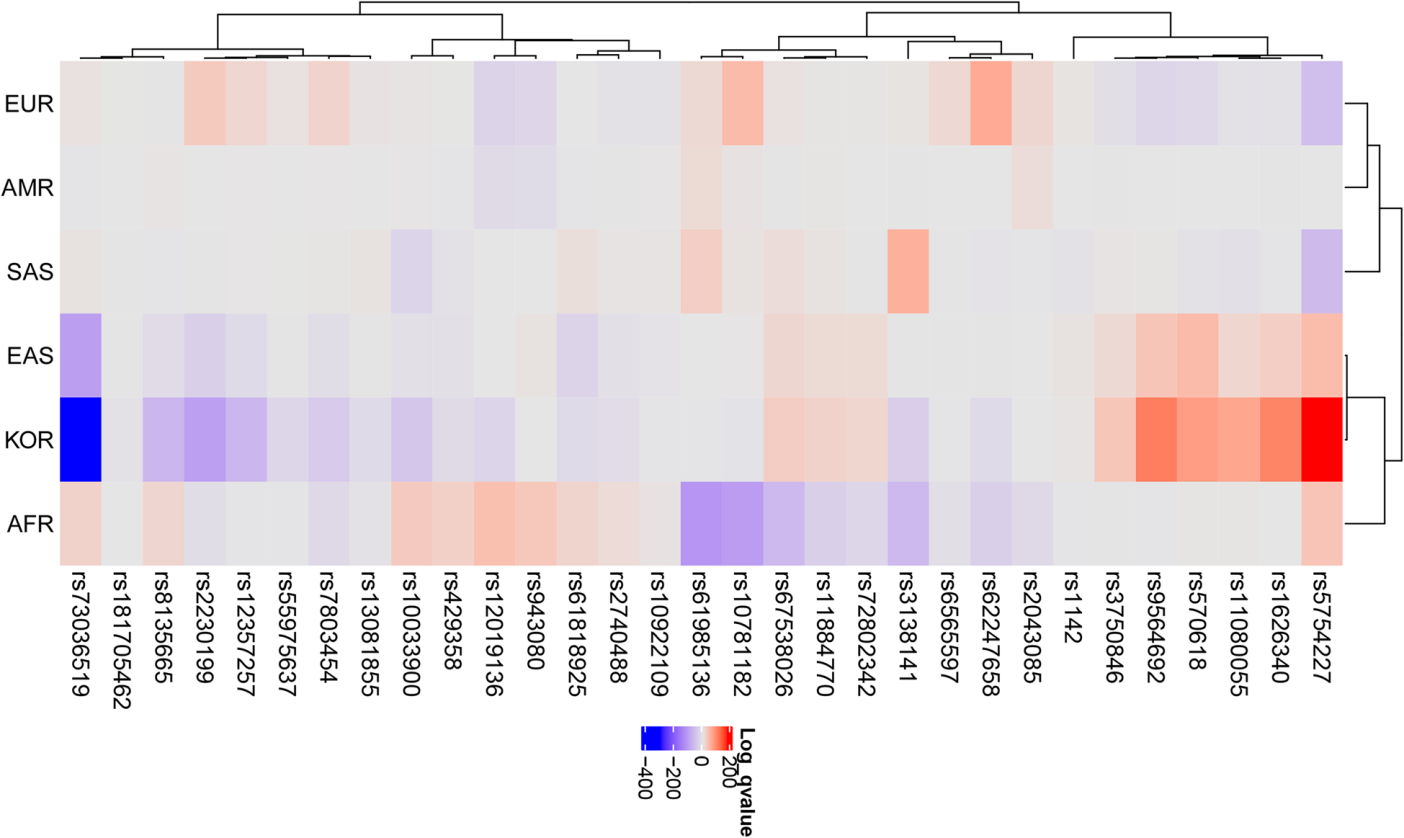
Heatmap generated using single nucleotide polymorphisms related to late age-related macular degeneration in the global population. Each row shows an SNP, and each column shows a continent. Red color indicates that the effect allele is enriched, whereas purple color indicates that the effect allele is depleted. AMR: American, EUR: European, SAS: South Asian, AFR: African, EAS: East Asian, KOR: Korean

Next, we compared the EAFs of East Asians and Koreans (**Supplementary Table S****2****and** Fig. 2). Although the EAF did not differ much among East Asians, 21 SNPs were enriched, 30 SNPs were depleted, and 87 SNPs were similar to those in Koreans. Moreover, the heatmap clearly showed that the main allele frequency pattern in East Asians in the 1000 Genomes Project was similar to that in Koreans; in contrast, few alleles showed the opposite allele frequency patterns between East Asians and Koreans.

### Patterns of late AMD-related SNPs in the global population and in east Asians

Thirty-one SNPs related to late AMD were selected from 138 AMD-related SNPs (Table 1), and a heatmap showing how significantly the EAF was enriched or depleted across the Koreans and continental groups (Fig. 2) with a log scale among 31 late AMD-related SNPs was generated. For Koreans, 10 AMD-related SNPs were significantly enriched, 19 SNPs were depleted, and two SNPs were similar to global EAFs. For example, rs11080055, located in the intronic region of the *TMEM97* gene, which encodes transmembrane protein 97, had A > C alleles; the C allele was detected in 49% of Europeans and 76% of Koreans. Additionally, rs1626340, located in the intergenic region of *TGFBR1/COL15A1*, which encodes collagen type XV alpha 1 chain, had G > A alleles; the A allele was detected in 21% of Europeans and 51% of Koreans. This SNP has been reported to be associated with various diseases, including colon cancer [25, 26]. rs5754227, located in the intronic region of *SYN3* which encodes synapsin-3 (a neuronal phosphoprotein associated with the cytoplasmic surface of synaptic vesicles), had T > C alleles; the C allele frequencies were 13% for Europeans and 67% for Koreans. Some SNPs were depleted in Koreans. For example, rs73036519, located in the intergenic region of the *EXOC3L2/MARK4* gene, which encodes a member of the microtubule affinity-regulating kinase family, had G > C alleles; the C allele frequencies were 29% for Europeans and 0% for Koreans. Hierarchical clustering tree analysis showed differences among the populations; Europeans, Americans, and South Asians were in one cluster, whereas Africans, East Asians, and Koreans were in another cluster (Fig. 2).

**Fig. 3:**
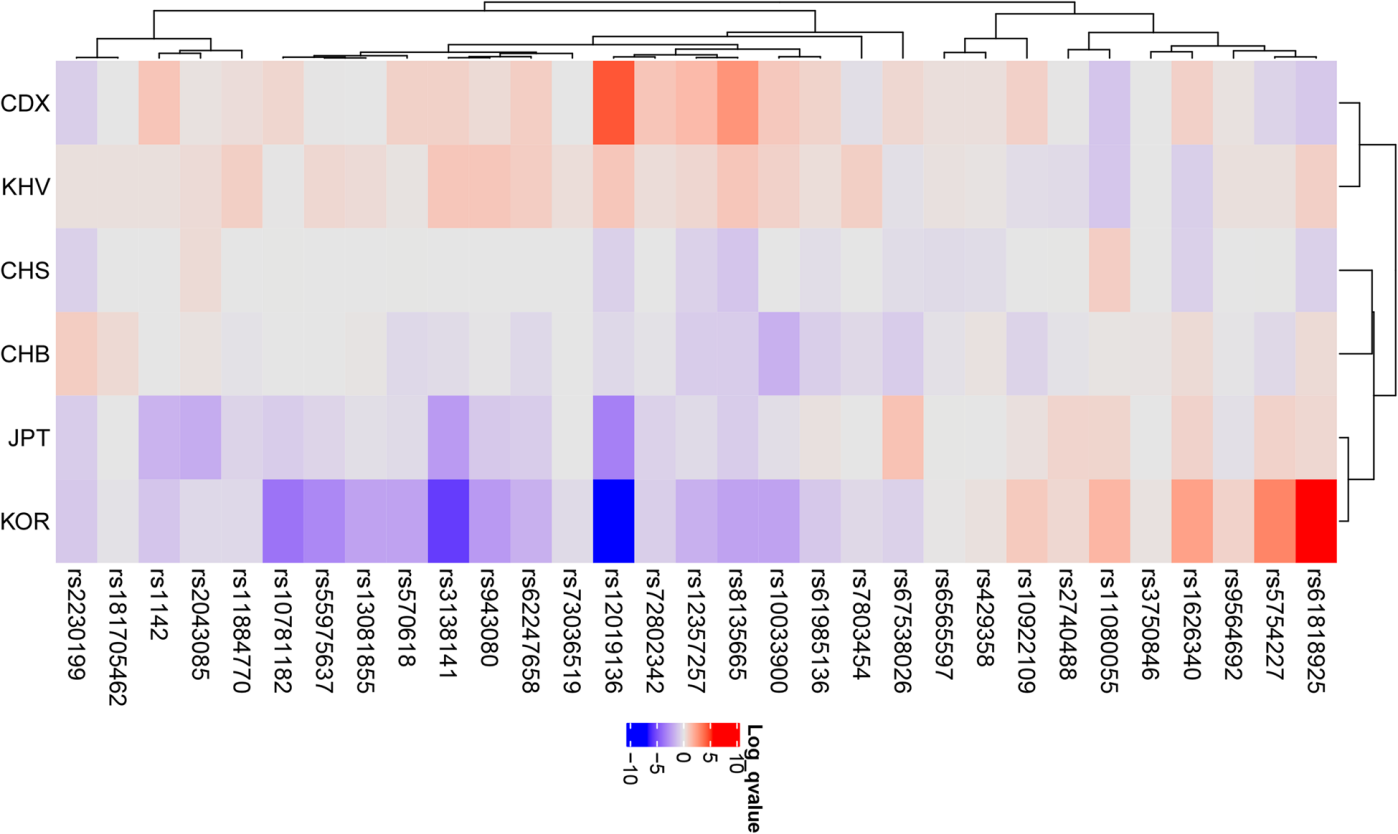
Heatmap generated using single nucleotide polymorphisms related to late age-related macular degeneration in the East Asian population. Each row shows an SNP, and each column shows a continent. Red color indicates that the effect allele is enriched, whereas purple color indicates that the effect allele is depleted. CDX: Chinese Dai in Xishuangbanna; CHB: Han Chinese in Beijing, China; CHS: Southern Han Chinese, China; JPT: Japanese in Tokyo, Japan; KOR: Korean in the Republic of Korea; KHV: Kinh in Ho Chi Minh City, Vietnam

In addition, heatmap analysis showed differences in EAFs across Koreans and East Asians (Table 2, Fig. 3). The rs61818925, located in the intergenic region of the *CFHR1* gene, which encodes complement factor H, had T > G alleles; the G allele frequencies were 33% for Chinese populations and 50% for Koreans. Notably, some SNPs were depleted in Koreans; for example, rs12019136, located in the intronic region of the *C3* gene, which encodes complement C3, had G > A alleles; the A allele frequencies were 21% for Vietnamese populations and 8% for Koreans. However, the EAFs did not differ substantially among East Asians; for Koreans, four SNPs were enriched, 11 SNPs were depleted, and 16 SNPs were similar to those in global East Asian populations.

**Table 2:** Effect allele frequencies (EAFs) of 31 late age-related macular degeneration related single nucleotide polymorphisms in East Asian groups including Koreans

SNP ID	Chr	Position	Function	Ref allele	Alt allele	Nearby/containing Gene	Global East Asian EAF	CHS EAF	CHS log_**10**_ ***P***	CDX EAF	CDX log_**10**_ ***P***	KHV EAF	KHV log_**10**_ ***P***	CHB EAF	CHB log_**10**_ ***P***	JPT EAF	JPT log_**10**_ ***P***	KOR EAF	KOR log_**10**_ ***P***
rs10033900	chr4	110,659,067	intergenic	T	C	CFI	0.39	0.39	0.00	0.45	0.92	0.43	0.65	0.31	−1.72	0.37	−0.24	0.34	−2.17
rs10781182	chr9	76,617,720	intergenic	T	G	MIR6130/RORB	0.49	0.51	0.01	0.54	0.50	0.49	0.02	0.50	0.00	0.41	−0.81	0.41	−3.69
rs10922109	chr1	196,704,632	intronic	C	A	CFH	0.44	0.46	0.00	0.51	0.67	0.40	−0.27	0.38	−0.57	0.47	0.19	0.47	0.89
rs11080055	chr17	26,649,724	intronic	A	C	TMEM97/VTN	0.72	0.80	0.77	0.63	−1.03	0.63	−0.99	0.74	0.08	0.77	0.54	0.76	1.50
rs1142	chr7	104,756,326	downstream	C	T	KMT2E/SRPK2	0.37	0.35	0.00	0.47	1.03	0.39	0.20	0.37	0.00	0.26	−1.61	0.34	−1.03
rs11884770	chr2	228,086,920	ncRNA_intronic	T	C	COL4A3	0.78	0.78	0.00	0.81	0.28	0.84	0.72	0.76	−0.13	0.73	−0.56	0.76	−0.43
rs12019136	chr19	5,835,677	intronic	G	A	C3	0.14	0.08	−0.69	0.29	4.20	0.21	0.99	0.10	−0.42	0.04	−3.29	0.08	−6.61
rs12357257	chr10	24,999,593	intronic	G	A	ARHGAP21	0.05	0.02	−0.65	0.11	1.37	0.08	0.47	0.02	−0.79	0.03	−0.35	0.03	−1.70
rs13081855	chr3	99,481,539	ncRNA_intronic	G	T	COL8A1/FILIP1L	0.05	0.04	−0.01	0.05	0.00	0.07	0.35	0.05	0.06	0.03	−0.22	0.03	−2.17
rs1626340	chr9	101,923,372	intergenic	G	A	TGFBR1/COL15A1	0.46	0.38	−0.69	0.53	0.67	0.38	−0.72	0.50	0.37	0.52	0.60	0.51	2.17
rs181705462	chr6	31,947,027	intronic	G	T	C2/CFB/SKIV2L	0.00	0.00	0.00	0.00	0.00	0.01	0.20	0.01	0.40	0.00	0.00	0.00	−0.13
rs2043085	chr15	58,680,954	intergenic	T	C	AQP9;LIPC	0.50	0.55	0.35	0.52	0.13	0.55	0.35	0.52	0.13	0.38	−1.85	0.48	−0.43
rs2230199	chr19	6,718,387	exonic	G	C	C3	0.00	0.00	−0.67	0.00	−0.75	0.01	0.20	0.01	0.79	0.00	−0.81	0.00	− 0.93
rs2740488	chr9	107,661,742	intronic	A	C	ABCA1	0.24	0.26	0.01	0.25	0.02	0.20	−0.35	0.22	−0.13	0.29	0.55	0.26	0.45
rs3138141	chr12	56,115,778	ncRNA_intronic	C	A	RDH5/CD63/MMP19	0.12	0.12	0.00	0.16	0.66	0.19	0.99	0.09	−0.33	0.04	−2.42	0.07	−5.43
rs3750846	chr10	124,215,565	intronic	T	C	ARMS2/HTRA1	0.41	0.39	−0.01	0.40	0.00	0.41	0.00	0.43	0.08	0.40	0.00	0.42	0.14
rs429358	chr19	45,411,941	exonic	T	C	APOE	0.09	0.06	−0.29	0.10	0.22	0.09	0.08	0.10	0.13	0.08	0.00	0.09	0.18
rs55975637	chr3	99,419,853	ncRNA_intronic	G	A	COL8A1	0.06	0.06	0.00	0.06	0.02	0.09	0.45	0.05	0.00	0.03	−0.53	0.03	−3.03
rs570618	chr1	196,657,064	intronic	T	G	CFH	0.95	0.96	0.01	0.98	0.66	0.96	0.11	0.93	−0.42	0.93	−0.35	0.92	−2.17
rs5754227	chr22	33,105,817	intronic	T	C	SYN3/TIMP3	0.61	0.63	0.01	0.55	−0.57	0.65	0.21	0.56	−0.40	0.67	0.62	0.67	2.97
rs61818925	chr1	196,815,450	intergenic	T	G	CFHR1	0.41	0.33	−0.69	0.32	−0.92	0.48	0.70	0.46	0.37	0.46	0.46	0.50	5.24
rs61985136	chr14	68,769,199	intronic	C	T	RAD51B	0.45	0.40	−0.26	0.51	0.59	0.49	0.27	0.38	−0.75	0.47	0.16	0.42	−0.93
rs62247658	chr3	64,715,155	ncRNA_intronic	C	T	ADAMTS9-AS2	0.24	0.25	0.00	0.31	0.75	0.31	0.78	0.19	−0.42	0.17	− 0.81	0.20	−1.70
rs6565597	chr17	79,526,821	intronic	C	T	NPLOC4/TSPAN10	0.21	0.17	−0.36	0.24	0.24	0.23	0.15	0.18	−0.15	0.22	0.01	0.21	0.03
rs67538026	chr19	1,031,438	intronic	C	T	CNN2	0.52	0.47	−0.29	0.57	0.46	0.49	−0.20	0.44	−0.79	0.62	1.12	0.50	−0.64
rs72802342	chr16	75,234,872	intergenic	C	A	CTRB2/CTRB1	0.13	0.12	0.00	0.20	1.03	0.16	0.30	0.11	−0.13	0.09	−0.65	0.11	−0.75
rs73036519	chr19	45,748,362	intergenic	G	C	EXOC3L2/MARK4	0.00	0.00	0.00	0.00	0.00	0.01	0.27	0.00	0.00	0.00	0.00	0.00	−0.35
rs7803454	chr7	99,991,548	intronic	C	T	PILRB/PILRA	0.03	0.03	0.00	0.02	−0.23	0.06	0.72	0.01	−0.40	0.03	0.00	0.02	−0.40
rs8135665	chr22	38,476,276	intronic	C	T	SLC16A8	0.14	0.09	−1.02	0.23	2.55	0.19	0.99	0.10	−0.79	0.10	−0.81	0.10	−2.17
rs943080	chr6	43,826,627	intergenic	C	T	VEGFA	0.73	0.73	0.00	0.76	0.37	0.79	0.99	0.72	−0.06	0.67	−0.92	0.67	−2.46
rs9564692	chr13	31,821,240	intronic	C	T	B3GALTL	0.71	0.71	0.00	0.73	0.14	0.73	0.20	0.70	−0.06	0.69	−0.20	0.73	0.63

*CHS* Southern Han Chinese, *China, CDX* Chinese Dai in Xishuangbanna, *China, KHV* Kinh in Ho Chi Minh City, Vietnam, CHB: Han Chinese in Beijing, China, *JPT* Japanese in Tokyo in 1000 genome project, *KOR* Korean Reference Genome data base, *Chr* chromosome, *EAF* effect allele frequency, *ref. allele* reference allele, *alt allele* alterative allele, *P*-value: adjusted Fischer’s test assuming 138 hypotheses, statistical significance was set at *P <* 0.05

### Genetic risk scores calculated using SNPs related to any AMD and late AMD

**Fig. 4:**
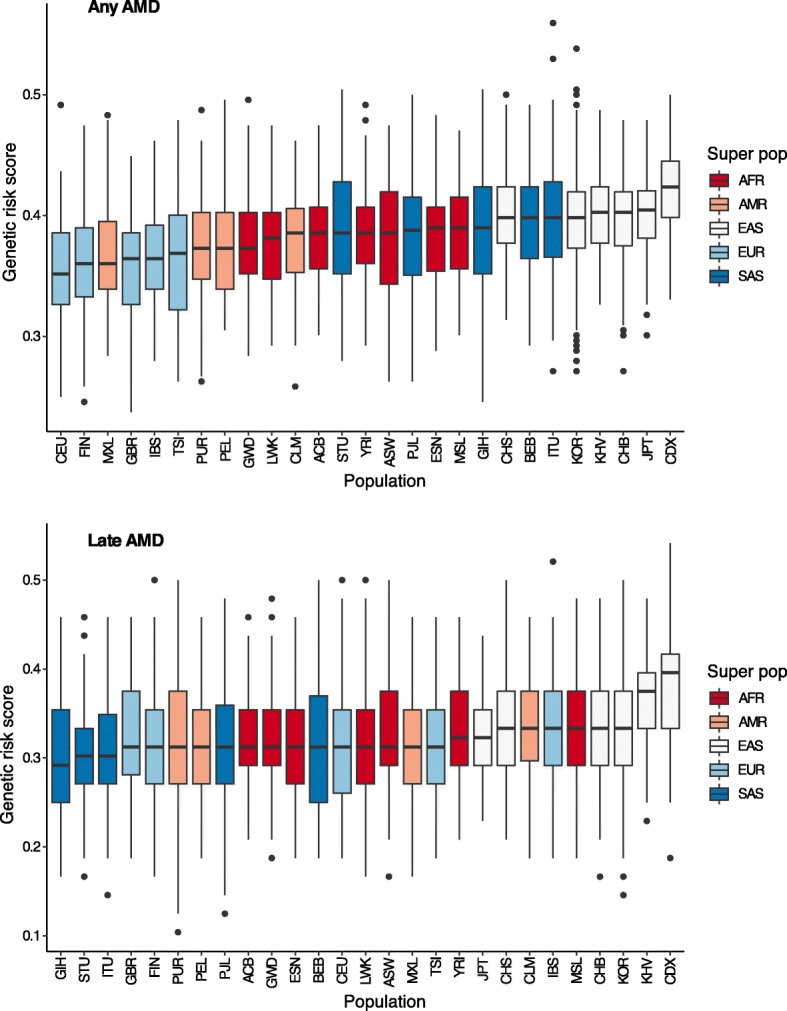
Genetic risk score calculations of age-related macular degeneration for any AMD or late AMD using related single nucleotide polymorphisms. ACB: African Caribbean in Barbados; ASW: African ancestry in the Southwest USA; BEB: Bengali in Bangladesh; CDX: Chinese Dai in Xishuangbanna; CEU: Utah residents with Northern and Western European ancestry; CHB: Han Chinese in Beijing, China; CHS: Southern Han Chinese, China; CLM: Colombian in Medellin, Colombia; ESN: Esan in Nigeria; FIN: Finnish in Finland; GBR: British in England and Scotland; GIH: Gujarati Indian in Houston, TX, USA; GWD: Gambian in Western Division, Gambia; IBS: Iberian populations in Spain; ITU: Indian Telugu in the UK; JPT: Japanese in Tokyo, Japan; KOR: Korean in the Republic of Korea; KHV: Kinh in Ho Chi Minh City, Vietnam; LWK: Luhya in Webuye, Kenya; MSL: Mende in Sierra Leone; MXL: Mexican ancestry in Los Angeles, CA, USA; PEL: Peruvian in Lima, Peru; PJL: Punjabi in Lahore, Pakistan; PUR: Puerto Rican in Puerto Rico; STU: Sri Lankan Tamil in the UK; TSI: Toscani in Italy; YRI: Yoruba in Ibadan, Nigeria

**Fig. 5:**
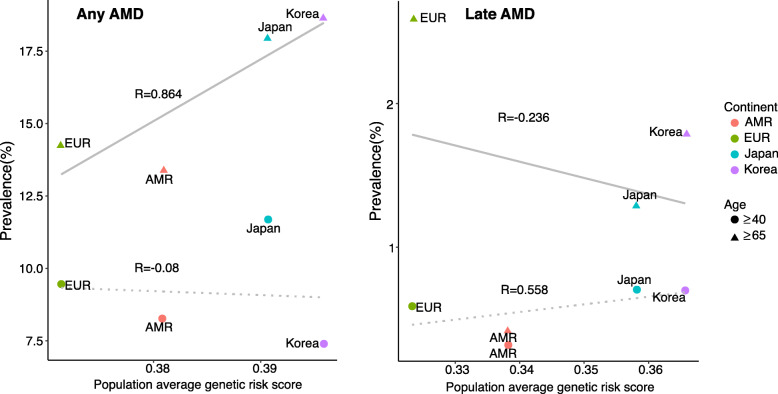
Correlation plots of the prevalence of age-related macular degeneration (any or late) and genetic risk score using related single nucleotide polymorphisms. The graphs on the left and right indicate correlations of prevalence and genetic risk scores with any AMD and late AMD, respectively. The dotted line indicates the relationship between genetic risk score and prevalence (circular shape) for people with AMD who are 40 years old or older, and the solid line indicates the relationship between genetic risk score and prevalence of AMD (triangular shape) for people 65 years of age or older. AMR: American, EUR: European

Next, we calculated the composite genetic risk scores based on copies of effect alleles at AMD-associated SNPs, with the assumption that allelic associations from most GWAS-identified variants could be replicated in non-European populations [27]. The genetic risk score of any AMD was highest in East Asians, followed by South Asians, Americans, and Europeans (Fig. 4). The prevalence of any AMD in individuals 65 years old or older was correlated with the population average genetic risk score (R = 0.864; Fig. 5). In addition, the genetic risk score of late AMD was highest in East Asians, followed by Europeans, Americans, and South Asians (Fig. 4). However, differences in genetic risk scores of late AMD were not greater than those of any AMD, as supported by meta-analysis data indicating that the prevalence of late AMD was similar among Europeans and Asians [7]. The prevalence of late AMD in individuals 40 years old or older was correlated with the population average genetic risk score (R = 0.558; Fig. 5). In addition, the PRS of any AMD or late AMD in East Asians was similar among Europeans and Asians (**Supplementary Fig. S****3**). The prevalence of any AMD in individuals 65 years old or older was correlated with the population PRS (R = 0.846; **Supplementary Fig. S****3**) and the prevalence of late AMD in individuals 40 years old or older was correlated with the population PRS (R = 0.572; **Supplementary Fig. S****3**).

## Discussion

The etiology of AMD is not fully understood; however, several hypotheses have demonstrated the involvement of genetics, inflammation, complement, lipids, and oxidative stress [28]; aging and family history are known risk factors for AMD [29]. Since the discovery of the *CFH* gene as a major AMD susceptibility gene in 2005, several studies using GWASs have reported various genetic risk loci [30–34]. Complement-related genes, such as complement H (*CFH*), complement factor I (*CFI*), and complement components *C2*, *C3*, and *C9*, are related to the pathogenesis of AMD [30]. *ARMS2/HTRA1* genes (rs10490924 and rs11200638) are risk factors for late AMD according to results of a Korean study [35]. In our study, we found that these SNPs showed higher prevalence rates in Koreans than in other races. Importantly, because the affected allele frequencies were high in their study, there were no problems with interpreting significance. The multi-hit threshold model of AMD pathogenesis [30] suggests that genes related to the complement pathway, immune response, lipid transport, extracellular matrix remodeling, angiogenesis, and cell survival are related to biologically relevant pathways. Therefore, race is expected to be one of important factor affecting AMD prevalence.

AMD prevalence differs among racial and ethnic groups [36]. According to a study by Wong and colleagues, early AMD occurs more frequently in individuals of European ancestry (11.2%) than in Asian individuals (6.8%), and any AMD was more common in populations of European ancestry (12.3%) than in those of Asian ancestry (7.4%) [2]. The occurrence of any AMD was markedly less common in populations of African ancestry [2, 21]. In a meta-analysis of AMD in Asians, the age-specific prevalence of late AMD in Asians was found to be comparable with that in Europeans; however, early AMD was found to be less common among Asians (6.8%) than among Europeans (8.8%) [7]. In our study, genetic risk scores of AMD were higher in Asians than in Europeans. Accordingly, our findings showed that the prevalence of AMD may be increased owing to genetic predisposition in East Asians, including Koreans. However, environmental factors, such as lifespan and eating habits, as well as differences in the penetrance of AMD-related genes may explain these results. For example, a study of Japanese immigrants in Brazil showed that the prevalence rates for early and late AMD were 13.4 and 1.3%, respectively, suggesting that genetic predisposition was affected by environmental factors [37]. In the Korea National Health and Nutrition Examination Survey, the prevalence rates of AMD were found to be 6.62% for any AMD, 6.02% for early AMD, and 0.6% for late AMD, similar to the prevalence rate of pooled Asian and European populations [23, 24]. In addition, according to a study of the projected number of people with AMD by region [2], global projected cases of any AMD in 2040 was the highest for Asian populations.

In a previous study, Fritsche and colleagues showed that the *ARMS2/HTRA1* gene plays a larger role in Asian populations than in European populations based on an analysis of the effects of allele frequencies of known AMD risk variants from 1000 Genomes data [30]. Thus, differences in genetic risk scores in Asians may favor late AMD, which could explain in prevalence of late AMD in Asian populations [7]. Any AMD was more prevalent in European populations than in Asian populations; however, Asian individuals may be more likely to develop late AMD than white individuals [36], consistent with the results of our study. Indeed, in our study, the country-wise AMD prevalence and genetic risk scores showed positive or negative correlations according age groups, suggesting that aging and environmental risk factors, such as smoking, may also play substantial roles in the development of AMD.

A major strength of our study was the inclusion of the large Korean whole-genome dataset (*n* = 1722), which reflected the allele frequency of SNPs related to any stage of AMD and to late AMD. Additionally, we did not systematically organize the new AMD cohort and analyze the effects; instead, we compared the 1000 Genomes Project data with AMD-related SNP data from the GWAS catalog. However, there were a few limitations to this study. First, the GWAS catalog contained data for which the risk allele was not clearly defined according to the minor allele frequency (MAF). However, we did not exclude these from in our study because the majority of MAFs were likely to be risk alleles; thus, removing all of the undefined alleles could result in inaccurate subgroup analysis. Further studies are needed for data curation of 50 undefined SNPs. To solve this problem, risk allele curation is necessary for the GWAS catalog based on the results of additional large population studies using AMD cohorts. Second, the statistical significance of EAF in Koreans was high and should be interpreted with caution because the Korean reference genome number was 1722 (whole genome data was 1099), which was very high; the genome number of populations belonging to the 1000 Genomes Project was approximately 100 (range: 61–113). In addition, there is a possibility that the difference in sequencing depth (30X in KRGDB vs. 4X in 1000 Genome Project) affects coverage. However, the authors expect fewer missing SNPs since the population frequency can be accurately found up to 1% through the combined calling method in the 1000 Genome Projects and the microarray consist of common SNPs. Third, our study analyzed data based on current knowledge; we did not detect new genetic loci or perform pathway analysis using cell culture and animal studies. Additionally, our findings should be interpreted while considering the fact that penetrance is variable, even when causative SNPs are present in specific individuals. Nevertheless, our results are expected to improve our understanding of the genetic etiology of AMD for ophthalmologists.

## Conclusion

Our study showed substantial population differentiation in allele frequencies for SNPs related to any AMD and late AMD. Based on the allele frequencies of these SNPs, the composite risk scores for AMD and late AMD for 26 ethnic groups in the 1000 Genomes Project and Koreans showed that East Asians, including Koreans, had a higher risk than Europeans for any AMD and late AMD. Finally, we observed differences in allele frequencies associated with SNPs related to AMD between Koreans and other races, which may explain the increased prevalence of AMD, predominantly in East Asians.

## Supplementary Information

**Additional file 1 Supplemental Figure 1** Heatmap generated using single nucleotide polymorphisms related to age-related macular degeneration in the global population. Each row shows an SNP, and each column shows a continent. Red color indicates that the effect allele is enriched, whereas purple color indicates that the effect allele is depleted. AMR: American, EUR: European, SAS: South Asian, AFR: African, EAS: East Asian, KOR: Korean.

**Additional file 2 Supplemental Figure 2** Heatmap generated using single nucleotide polymorphisms related to age-related macular degeneration in East Asian populations. Each row shows an SNP, and each column shows a country. Red color indicates that the effect allele is enriched, whereas purple color indicates that the effect allele is depleted. CDX: Chinese Dai in Xishuangbanna; CHB: Han Chinese in Beijing, China; CHS: Southern Han Chinese, China; JPT: Japanese in Tokyo, Japan; KOR: Korean in the Republic of Korea; KHV: Kinh in Ho Chi Minh City, Vietnam.

**Additional file 3 Supplemental Figure 3** Polygenic risk score calculations of age-related macular degeneration for any AMD or late AMD using related single nucleotide polymorphisms and correlation plots of the prevalence of age-related macular degeneration (any or late) and polygenic risk score

**Additional file 4 Supplemental Table 1** Effect allele frequencies (EAFs) of any age-related macular degeneration related single nucleotide polymorphisms in continental groups, including Koreans.

**Additional file 5 Supplemental Table 2 **Effect allele frequencies (EAFs) of any age-related macular degeneration-related single nucleotide polymorphisms in East Asian groups, including Koreans.

## Abbreviations

AMD: Age-related macular degeneration
CNV: Choroidal neovascularization
C3: Complement component 3
CFH: Complement factor H
CFI: Complement factor I
EAFs: Effect allele frequencies
GWASs: Genome-wide association studies
GA: Geographic atrophy
IRB: Institutional Review Board
KRGDB: Korean Reference Genome Database
MAF: Minor allele frequency
NHGRI-EBI: National Human Genome Research Institute-European Bioinformatics Institute
PRS: Polygenic risk score
SNPs: Single nucleotide polymorphisms
