# Supplementary figures and images for: Comparison of risk allele frequencies of single nucleotide polymorphisms associated with age-related macular degeneration in different ethnic groups
Source: BMC Ophthalmol. 2021 Feb 22;21:97. doi: 10.1186/s12886-021-01830-9 (PMC7898441; doi:10.1186/s12886-021-01830-9)

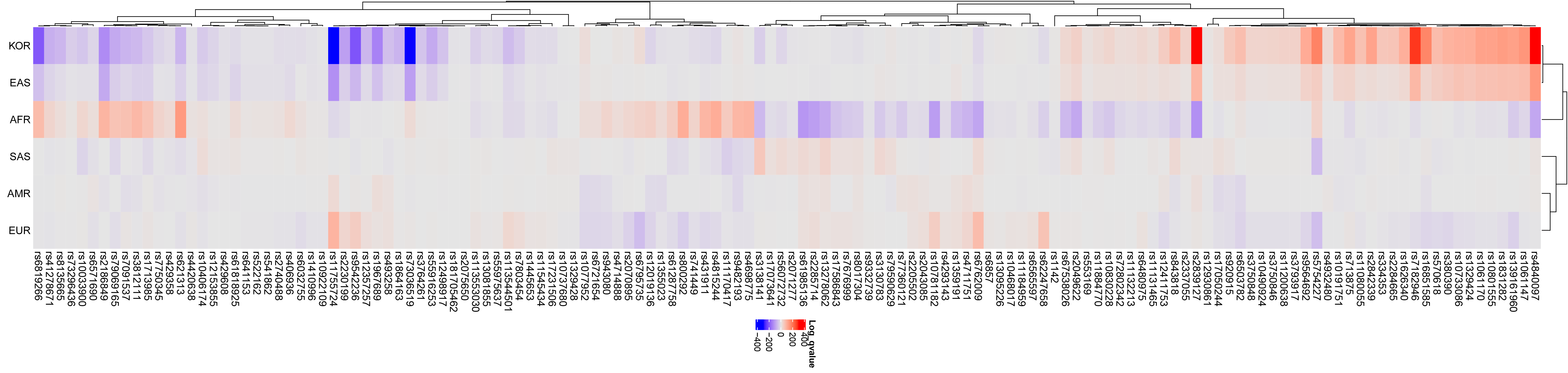

Supplement: Supplementary file 1 — Additional file 1 Supplemental Figure 1 Heatmap generated using single nucleotide polymorphisms related to age-related macular degeneration in the global population. Each row shows an SNP, and each column shows a continent. Red color indicates that the effect allele is enriched, whereas purple color indicates that the effect allele is depleted. AMR: American, EUR: European, SAS: South Asian, AFR: African, EAS: East Asian, KOR: Korean. [file 12886_2021_1830_MOESM1_ESM.tif]

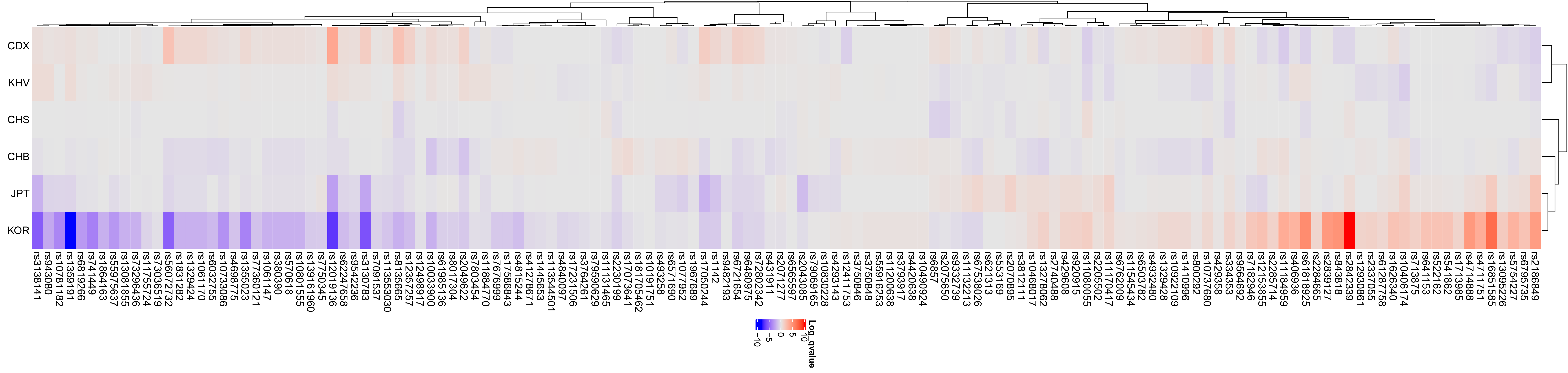

Supplement: Supplementary file 2 — Additional file 2 Supplemental Figure 2 Heatmap generated using single nucleotide polymorphisms related to age-related macular degeneration in East Asian populations. Each row shows an SNP, and each column shows a country. Red color indicates that the effect allele is enriched, whereas purple color indicates that the effect allele is depleted. CDX: Chinese Dai in Xishuangbanna; CHB: Han Chinese in Beijing, China; CHS: Southern Han Chinese, China; JPT: Japanese in Tokyo, Japan; KOR: Korean in the Republic of Korea; KHV: Kinh in Ho Chi Minh City, Vietnam. [file 12886_2021_1830_MOESM2_ESM.tif]

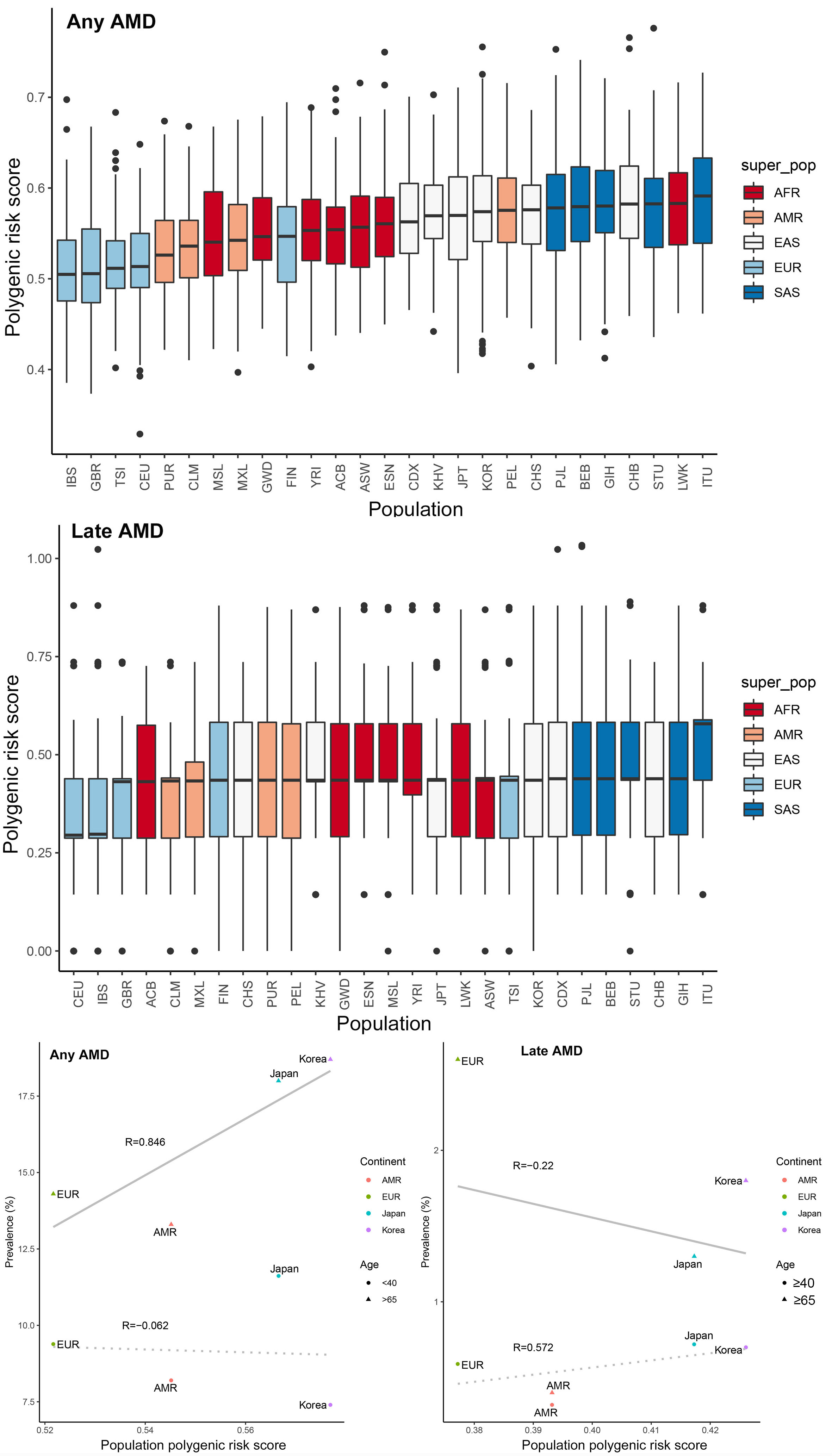

Supplement: Supplementary file 3 — Additional file 3 Supplemental Figure 3 Polygenic risk score calculations of age-related macular degeneration for any AMD or late AMD using related single nucleotide polymorphisms and correlation plots of the prevalence of age-related macular degeneration (any or late) and polygenic risk score [file 12886_2021_1830_MOESM3_ESM.jpg]
